# Ensemble of Chaotic and Naive Approaches for Performance Enhancement in Video Encryption

**DOI:** 10.1155/2015/458272

**Published:** 2015-10-13

**Authors:** Jeyamala Chandrasekaran, S. J. Thiruvengadam

**Affiliations:** ^1^Department of Information Technology, Thiagarajar College of Engineering, Madurai 625015, India; ^2^Department of Electronics and Communication Engineering, Thiagarajar College of Engineering, Madurai 625015, India

## Abstract

Owing to the growth of high performance network technologies, multimedia applications over the Internet are increasing exponentially. Applications like video conferencing, video-on-demand, and pay-per-view depend upon encryption algorithms for providing confidentiality. Video communication is characterized by distinct features such as large volume, high redundancy between adjacent frames, video codec compliance, syntax compliance, and application specific requirements. Naive approaches for video encryption encrypt the entire video stream with conventional text based cryptographic algorithms. Although naive approaches are the most secure for video encryption, the computational cost associated with them is very high. This research work aims at enhancing the speed of naive approaches through chaos based S-box design. Chaotic equations are popularly known for randomness, extreme sensitivity to initial conditions, and ergodicity. The proposed methodology employs two-dimensional discrete Henon map for (i) generation of dynamic and key-dependent S-box that could be integrated with symmetric algorithms like Blowfish and Data Encryption Standard (DES) and (ii) generation of one-time keys for simple substitution ciphers. The proposed design is tested for randomness, nonlinearity, avalanche effect, bit independence criterion, and key sensitivity. Experimental results confirm that chaos based S-box design and key generation significantly reduce the computational cost of video encryption with no compromise in security.

## 1. Introduction

The advancements in networks and communication have promoted the usage of digital multimedia data in the field of education, commerce, defense, and entertainment. Usage of video applications such as video messaging, video conferencing, video surveillance, and Internet video sites such as YouTube is becoming increasingly popular and pervasive. Telemedicine is an application of clinical medicine and information technology where consultation, medical procedures, and examinations are performed remotely using interactive audiovisual media. These applications generate and transmit large amount of sensitive data in a resource constrained environment characterized by lower bandwidth, frequent packet drops, quality of service concerns, and limited computational resources of the communicating devices. The increasing use of video based applications demands not only secure algorithms for transmission and storage of videos but also efficient algorithms to reduce computational overhead.

Confidentiality for multimedia data is offered by means of encryption, in which the legitimate users can only gain access to data with the secret keys possessed by them. Video encryption algorithms are classified based on the way of encrypting data. Fully Layered Encryption (Naive Encryption) considers the whole content of video stream as a sequence of bits and encrypts with text based symmetric 3 algorithms such as Advanced Encryption Standard (AES), Blowfish, and Triple DES and so forth [1, 2]. As these algorithms have no strong evidences of cryptanalysis reports, they provide complete security to the whole multimedia stream. However, because of the complexity involved in key generation and encryption, delay increases and overload will be unacceptable for real time processing.

To increase the encryption efficiency, a large number of permutation and selective video encryption strategies are proposed in the literature. Permutation based video encryption algorithms scramble the bytes within every frame of a video stream. In Zig-Zag permutation [3], an individual 8 × 8 block is mapped onto 1 × 64 vector by using a random permutation list. Shi and Bhargava [4] proposed a methodology to save computational time by combining MPEG compression and data encryption at the same time. As MPEG compression rate depends upon Huffman codeword list, the same codeword list is used as a secret key for permutation. Permutation based algorithms are vulnerable to known plain text attack [5], because by comparing the ciphered frames with the original frames, the permutation list can be easily figured out.

Selective encryption algorithms encrypt only selective subset of bytes within video frames to reduce the volume of data to be encrypted and at the same time to preserve a sufficient level of security. Meyer and Gadegast [6] proposed a scheme in which RSA or DES was used to encrypt MPEG video stream at four levels of security: (i) encrypting all stream headers, (ii) encrypting all stream headers and all DC and lower AC coefficients of intracoded blocks, (iii) encrypting I-frames and all I-blocks in P and B frames, and (iv) encrypting all the bit streams. However, special encoder and decoder are required to read unencrypted stream. In Aegis scheme proposed by Spanos and Maples [7], the intraframes, video stream header, and the ISO 32-bit end code of the MPEG stream are encrypted using DES in Cipher Block Chaining (CBC) mode, while the P and B frames are left unencrypted. The limitation of this idea is that the stream headers are predictable and broken by plaintext-ciphertext pairs [8]. Socek et al. [9] proposed a video encryption algorithm that securely encrypts the video stream but also preserves data correlation in the video sequence. An improvised H.264/AVC comprehensive video encryption scheme is proposed by Wang et al. [10] in which the intraprediction mode, motion vector difference, and quantization coefficients are encrypted and the encryption keys are generated based on the cryptographic hash function.

From the literature, it can be inferred that there is no scheme that fits all applications. Permutation and selective encryption algorithms can reduce the computational overhead, but for complete and provable security of video in sensitive applications such as telemedicine, military, and business meetings, the entire video stream needs to be encrypted. Hence, development of video encryption algorithms encrypting the entire video stream at low computational cost is inevitable.

The proposed system encrypts the entire stream of video data but with a significant reduction in computational time through the application of Henon map. Henon map is employed in video encryption to address two levels of security: (i) ensemble of dynamic and key-dependent S-box with symmetric algorithms (Blowfish and DES) and (ii) generation of one-time keys for simple substitution ciphers.

## 2. Background

### 2.1. S-Box

In cryptography, an S-box is a basic component of symmetric key algorithms, which performs substitution. It is typically used to establish a nonlinear relationship between the key and the cipher text. An S-box should exhibit cryptographic properties such as bijection, strict avalanche, output bits independence, and equiprobable input/output XOR distribution. The input to an S-box could be an “*n*” bit word, but the output can be an “*m*” bit word, where “*m*” and “*n*” are not necessarily the same. An S-box can also be keyed or keyless and linear or nonlinear. S-boxes help the block ciphers to exhibit two important characteristics, namely, diffusion and confusion. The idea of diffusion is to hide the relationship between the plain text and the cipher text, which will frustrate the adversary who uses cipher text statistics to find the plain text. The idea of confusion is to hide the relationship between the cipher text and the key, which will frustrate the adversary who uses cipher text to find the key.

### 2.2. Analysis of S-Box Design in Symmetric Cryptosystems

Eight S-boxes are used in DES, in which each S-box is constructed as a table of four rows and sixteen columns [11]. The design of S-box in DES is not open, not key-dependent, and not dynamic, which could serve as a point of vulnerability. In Blowfish, four S-boxes are used each of which is constructed as a one-dimensional array of 256 elements [12]. Even though the S-box values are key-dependent, 521 Blowfish encryptions are required to generate all values thereby leading to a significant increase in computational time. The S-boxes of AES are prone to being attacked and hard to mask for its nonlinear characteristic. In addition, large amounts of circuit resources in chips and power consumption are involved in protecting S-boxes against power analysis [13]. Many researchers have proposed alternative design of S-boxes to address the above-mentioned limitations. The following four approaches are normally followed in S-box design [14].

- Random: pseudorandom numbers are used to generate the entries in the S-boxes.
- Random with testing: the S-box entries are chosen randomly and tested against various criteria and the values that do not pass are not considered.
- Human-made: the S-boxes of DES are designed in this mode, where the entries are filled manually. However, the approach is difficult to carry out for large S-boxes.
- Math-made: S-boxes are generated by using mathematical principles, and hence they have proven security against linear and differential cryptanalysis. Use of chaotic equations is one such mathematical way of S-box design.

### 2.3. Related Works

Discretized chaotic maps are used for generating dynamic and cryptographically strong S-boxes by Tang and Liao [15]. The cryptographic properties such as bijection, nonlinearity, strict avalanche, output bits independence, and equiprobable input/output XOR distribution of these S-boxes were analyzed in detail. A method for designing S-box based on chaos and genetic algorithm is proposed by Wang et al. [16]. The problem of S-box generation is converted into a Travelling Salesman Problem (TSP). A stronger S-box is obtained because of the complete utilization of traits of chaotic map and evolution process. Attempts have also been made to design strong S-boxes based on chaotic Lorenz system [17] and Tangent delay for elliptic cavity chaotic sequence [18]. Lambić [19] has used an idea of ensemble of chaotic maps and composition method to generate random and bijective S-boxes. It is based on compositions of S-boxes from a fixed starting set. The sequence of the indices of starting S-boxes used is obtained by using chaotic maps. Chandrasekaran et al. [20] have proposed a method based on logistic map for S-box generation and reported the reduction in computational costs for text and image encryption on integration with Blowfish algorithm.

Henon map has been used for pseudorandom number generation [21] and for encryption of satellite imagery [22]. Most of the research articles have analyzed the effect of one-dimensional chaotic system for S-box generation. This research work employs two-dimensional discrete Henon map in construction of S-boxes and in generation of one-time keys for video encryption, as Henon map offers better pseudo randomness than logistic maps [23].

### 2.4. Henon Map

The random-like, unpredictable dynamics and simplicity in realization of chaotic systems make them suitable for cryptographic applications. The Henon map is a two-dimensional discrete dynamical system introduced by Michael Henon as a simplified model of Lorenz system [24]. Henon map takes a point (*x*_*n*_,  *y*_*n*_) in the plane and maps it onto a new point (*x*_*n*+1_,  *y*_*n*+1_) as defined by the equations

(1) xn+1=yn+1+1−αxn2yn+1=βxn.

The desired statistical properties can be obtained from the generated values if the input values are as follows. “*α*” can be in the range of 1.16 to 1.41. “*β*” can be in the range of 0.2 to 0.3. “*x*_0_” can be in the range of −1 to 1. “*y*_0_” can be in the range of −0.35 to 0.35. Skip value can be in the range of 80 to 1000.

**Figure 1:**
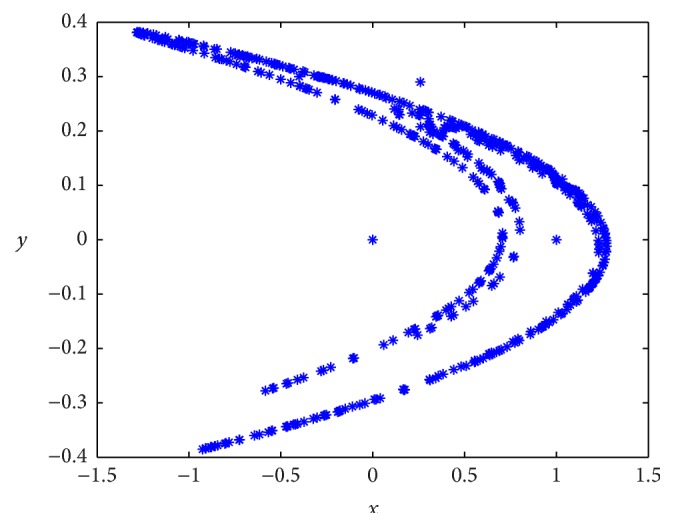
Chaotic behavior of Henon map.

Henon map is found to exhibit good chaotic behavior for values *α* = 1.4 and *β* = 0.3, as shown in Figure 1.

A minute variation in the initial parameters even in ten-millionth place value of the chaotic systems could yield widely divergent results. In any cryptographic application, the property of confusion aims to make the relationship between statistics of cipher and key as complex as possible to thwart attempts to discover the key. The property of diffusion aims to make the statistical relationship between the input and cipher as complex as possible. The extreme sensitiveness to initial conditions of the Henon map makes it suitable for cryptographic applications due to the enhanced confusion and diffusion properties.

## 3. Application of Henon Maps in Proposed S-Box Design

Certain applications such as business and military meetings require a very high level of security. Extreme level of security could be achieved only by naive approaches of video encryption but with high computational costs. In order to increase the encryption speed and to reduce the computational cost of symmetric algorithms, the proposed S-box design using Henon maps can be integrated into the chosen symmetric algorithms.

### 3.1. Implementation Procedure

The various steps involved in integration of the proposed S-box design with symmetric algorithms including Blowfish and DES are as follows.

**Step 1 (exchange of secret parameters).**

For generation of S-boxes, the following parameters are to be exchanged using the public keys of the communicating entities or through a secret channel.

$P_{1}$: The parameters of the Henon map  *α*,  *β* and seed values *x*_0_,  *y*_0_.
$P_{2}$: The number for decimal places of the mantissa that are to be supported by the calculating machine.
$P_{3}$: The number of iterations after which the first value is to be picked for generating keys.
$P_{4}$: The skip value to be maintained for picking successive values thereafter.

**Step 2 (encoding of chaotic values into binary sequence).**

(1) Iterate the two-dimensional equations of the Henon map for a predefined number of times, which is specific to the crypto algorithm chosen for encryption. Process the result in two one-dimensional arrays** x** and** y** of length *d* = *wm*/2. The number of iterations (*N*_*c*_) is given by

(2) $$\begin{array}{c} N_{c}=\frac{1}{2}\left(\;wmv\;\right), \end{array}$$

where “*w*” represents the word length of S-box elements. “*m*” represents the total number of S-box elements. “*v*” represents the skip value to be maintained for picking up successive values in iterations of Henon map equations.

**Figure 2:**
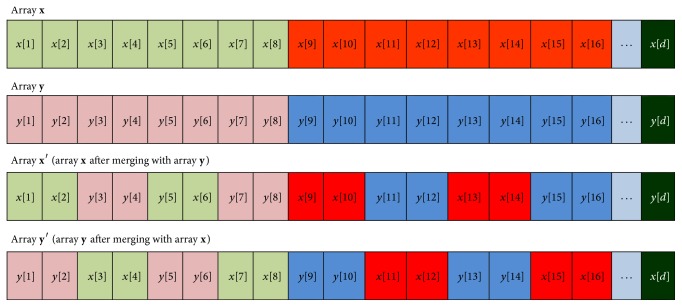
Encoding of chaotic values into binary sequence.

(2) Split the arrays **x** and **y** into subarrays of length equal to the word length (*w*) of the S-box elements.

(3) Mix the individual subarrays with each other at predefined crossover points as illustrated in Figure 2.

(4) Calculate the medians (*τ*_*x*_′ and *τ*_*y*_′) of each individual array of **x**′ and **y**′.

(5) To generate a binary sequence, compare each element of *x*′ and *y*′ with its median, *τ*_*x*_′ and *τ*_*y*_′, respectively. If *x*[*i*] is greater than *τ*_*x*_′, encode as “1”; else encode as “0.” If *y*[*i*] is greater than *τ*_*y*_′, encode as “1”; else encode as “0.”

(6) Partition each binary sequence across the word length and use alternatively to generate the individual S-box elements.

A sample 8-bit S-box with eight rows and eight columns (64 elements) is illustrated in (3). It could be inferred that the S-box entries are almost random and nonlinear:

(3) 2371411522282307810914113487101591845713486158102251571522242162179912918299101251001021022301029715314291021551028118257134101100109152216921714321523654831181015799108.

**Step 3 (video encryption).**

Let *V* be the video to be encrypted consisting of frames *F*_1_, *F*_2_, *F*_3_,…, *F*_*z*_. The S-boxes generated are integrated with the Feistel cipher structure of both Blowfish and DES to perform encryption. The entire video stream is treated as a sequence of bits and is completely encrypted with the corresponding crypto algorithm. A detailed description of Blowfish and DES can be found in [11] and [12], respectively.

### 3.2. Experimental Results and Discussion

#### 3.2.1. Performance Enhancement

Integration of the proposed S-box design with DES aims to enhance security through dynamic and key-dependent S-boxes. Existing S-box design of DES is static and hence it is possible for an opponent to perform cryptanalysis by exploiting its weaknesses [25]. Cryptanalysis of DES mainly focuses on the construction of eight S-boxes. The present work increases the resistivity of DES against cryptanalysis. The significant advantage of key-dependent S-boxes is that it is computationally impossible to analyze the S-box values ahead of time to look for weaknesses.

Existing Blowfish algorithm requires 521 encryptions for generation of P-arrays and S-boxes. The proposed S-box design when integrated with Blowfish results in significant reduction of time by eliminating the 521 encryptions. Thus, the proposed S-box design makes Blowfish suitable for memory limited and low powered devices with no compromise in security.

#### 3.2.2. Nonlinearity

Nonlinearity is the major requirement of any S-box design. The proposed design of S-boxes is tested for nonlinearity against different criteria [25] as given below. As a sample, the S-box generated for integration with DES is used as a test case and is shown in (4)

(4) 00010203040506070809101112131415001567209815063121116301102127171143141541114860213140810334314871401303801110141545926133817.

The S-boxes thus generated are tested against various criteria specified for quality checks and the results are as follows.

**Criterion 1 (no output bit of any S-box should be too close to a linear function of the input bits).**

**Figure 3:**
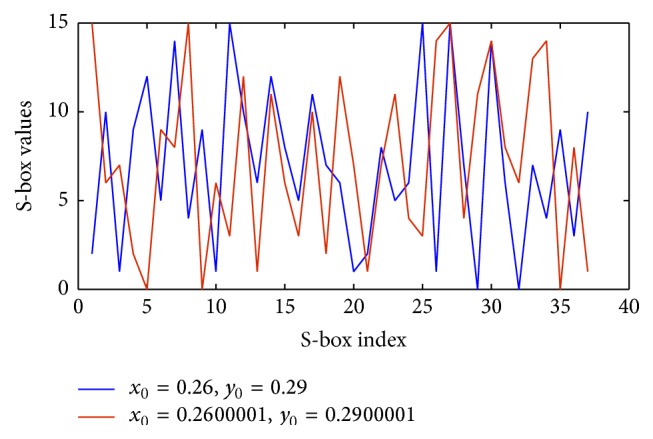
Nonlinear relations between input and output bits.

The relation between the input and output bits is plotted in Figure 3 for different seed values. The graph depicts a highly nonlinear relationship between the input and output bits.

**Criterion 2 (if two inputs to an S-box differ in exactly one bit, the outputs must differ in at least two bits).**

For the given inputs [110000,110001] and [100000,110000], which differ by one bit, the corresponding outputs are [0011,1000] and [1101, 1000] which differ by at least two bits.

**Criterion 3 (if two inputs to an S-box differ in the two middle bits exactly, the outputs must differ in at least two bits).**

For the given inputs [001100, 000000] and [110011, 111111], which differ by two middle bits exactly, the corresponding outputs are [1001,1111] and [1001, 0111] which differ by at least two bits.

**Criterion 4 (if two inputs to an S-box differ in their first two bits and are identical in their last two bits, the two outputs must not be the same).**

For the given inputs [110011, 000011] and [100000, 010000], which differ in two middle bits exactly, the corresponding outputs are [1001,0010] and [1101, 1010] which differ by at least two bits.

The proposed S-box design has been tested for the above four criteria for 30 test cases drawn from 10 samples of S-boxes. About 99% of the test cases were found satisfactory with respect to all criteria.

#### 3.2.3. Key Sensitivity Analysis

For a good cryptosystem, the output should vary significantly by a larger number of bits for minor changes in input. A sample of generated and encoded values in decimal format for minor changes in seed values of Henon map is shown in Table 1. It can be inferred that the generated S-box values for varying inputs that differ by a minor value result in significant changes in the output, thus making crypt analysis practically infeasible.

#### 3.2.4. Avalanche Effect

A desirable property of any cryptographic algorithm is that the key space should be discretized in such a way that two ciphers encrypted by two slightly different keys *k*_1_, *k*_2_ ∈ *K* should be completely different. The proposed methodology has been examined with Blowfish encryption for text inputs with a difference of 1 bit retaining all the other inputs to be similar. If a function is to satisfy the strict avalanche criterion, then each of its output bits should change with a probability of one half whenever a single input bit is complemented. The deviation has been analysed in each round of Blowfish encryption for the input parameters, Key = 0 × 78906543,   *X*_0_ = 0.26, *Y*_0_ = 0.29, skip value = 100 and slightly differing plain text input 1 = 0 × 1234876509874563, and plain text input 2 = 0 × 1234876509874562. It can be observed from Table 2 that the output of each round significantly differs by large number of bits thereby exhibiting good avalanche effect.

## 4. Application of Henon Maps in Generation of Random Keys for Simple Substitution Cipher

Certain applications like pay TV require medium level of security but high speed for video encryption. To facilitate this, the chaotic values generated by the Henon map are encoded as one-time keys. As the encryption and decryption involve a simple and reversible XOR operation of keys with all the pixels of every frame, the time taken for encryption and decryption is significantly reduced.

### 4.1. Implementation Procedure

The steps involved in generation of random keys for simple substitution cipher operating in counter mode of operation are as follows.

**Step 1 (exchange of secret parameters).**

In addition to the exchange of secret parameters (*P*_1_,   *P*_2_, *P*_3_,   *P*_4_) as mentioned in Section 3.1 (Step 1), another parameter for increment of seed value (*P*_5_) is also exchanged. For random generation of keys, the seed values *X*_0_ and *Y*_0_ are incremented by the value of *P*_5_ during encryption of every frame.

**Step 2 (encoding of chaotic values into binary sequence).**

(1) Iterate the Henon map for a predefined number of times, which directly depends upon number of frames and count of pixels in each frame. The number of iterations (*N*_*k*_) is given by

(5) $$\begin{array}{c} N_{k}=\frac{1}{2}\left(\;zlbv\;\right), \end{array}$$

where “*z*” represents the number of frames in the video sequence. “*l*” represents the number of pixels in every frame. “*b*” represents the number of bits used for pixel representation. “*v*” represents the skip value to be maintained for picking up successive values in iterations of Henon map equations.

(2) Create a key table consisting of all possible 2^*b*^ keys, which is sequentially arranged and indexed.

(3) Encode the chaotic values generated to the indices of the key table using the same procedure as described in Section 3.1 (Step 2). Identify the key for encryption from the corresponding index of the key table.

**Step 3 (video encryption).**

Let *V* be the video to be encrypted consisting of frames *F*_1_, *F*_2_, *F*_3_,…, *F*_*z*_. The number of pixels in every frame is represented by “*l*” and “*b*” bits are used for every pixel representation.

**Figure 4:**
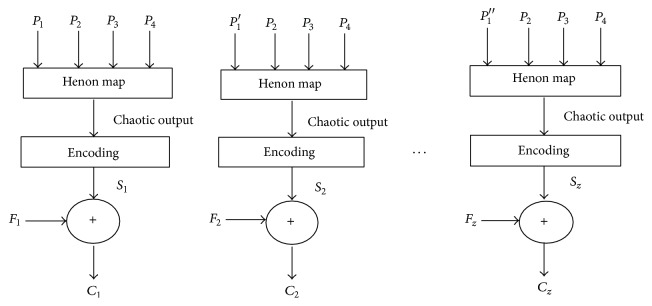
Miniature substitution cipher with counter mode of operation.

(1) Perform XOR operation on every pixel (*R*_1_, *R*_2_,…, *R*_*l*_) of the frame (*F*_1_) with the keys (*K*_1_, *K*_2_,…, *K*_*l*_) in keyset (*S*_1_) to produce the ciphered frame (*C*_1_) as illustrated in Figure 4.
(2) Update the seed (*P*_1_) using the increment parameter (*P*_5_) to generate new random keys for encryption of every frame, thereby making the crypto system as strong as one-time pads. To enhance the speed of encryption, the key generation can be done as a preprocessing step prior to encryption.

### 4.2. Experimental Results and Discussion

The key generation algorithm has been implemented in MATLAB in Microsoft Windows environment and is tested with various video formats. The strength of the proposed design and its suitability for video encryption have been tested for key space, key sensitivity, resistance against statistical attacks, randomness, and computational time. The experimental results are demonstrated for “dfs.avi” file and its properties are enlisted in Table 3.

**Figure 5:**
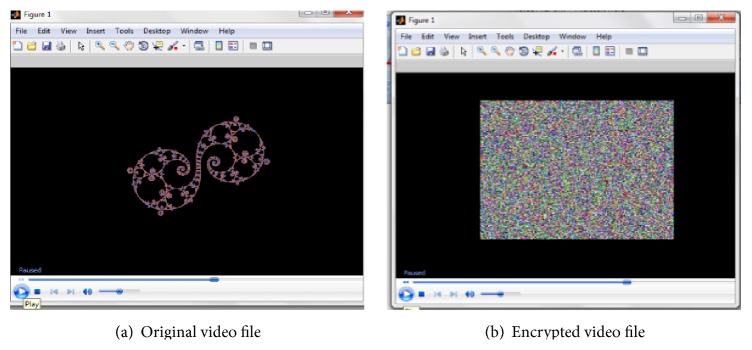
Encryption of video file.

**Figure 6:**
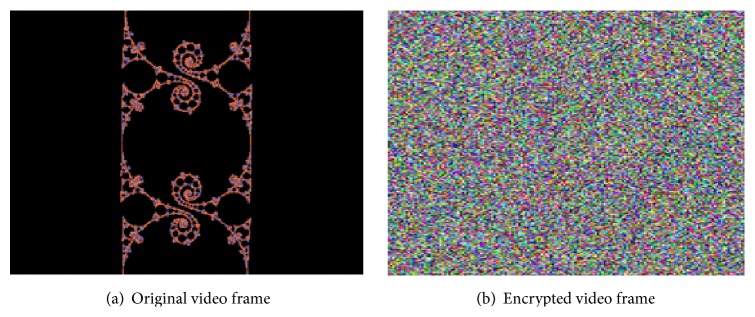
Encryption of a single frame of “dfs.avi” video.

The results of encrypting the video file and an individual frame are illustrated in Figures 5 and 6, respectively. It can be inferred that encryption with the proposed scheme provides complete visual degradation, thus providing no clue to deploy statistical and differential attacks.

#### 4.2.1. Key Space Analysis

Attempt to recover the original data by trying out all possible keys is called Brute force attack. The strength of any cryptographic algorithm depends on the size of its key space to make Brute force attack infeasible. The values of S-boxes depend on the initial seed and its related parameters associated with the chaotic equation. The initial seed “*X*_0_” is in the range of −1 to 1 and “*Y*_0_” is in the range of −0.35 to 0.35. The key space also depends upon the number of decimal places of the mantissa that are supported by the calculating machine which is approximately infinitely large, thus making Brute force attack computationally infeasible.

#### 4.2.2. Resistance against Statistical Attacks

*(A) Correlation Coefficient*. A high level of correlation exists among pixels in adjacent frames of a video and in order to make a video encryption algorithm resistant towards crypt analysis, the correlation between the adjacent frames of the ciphered video must be minimized. The correlation coefficient (*ρ*) is calculated by

(6) ρ=G∑j=1Gpjqj−∑j=1Gpj∑j=1GqjG∑pj2−∑j=1Gqj2G∑pj2−∑j=1Gqj2,

where *p* and *q* are the intensity values of two pixels in the same position of adjacent frames and *G* represents the total number of pixels in a frame. From Table 4 it is inferred that there exists negligible correlation between plain and ciphered images, thereby providing no clue for statistical crypt analysis.

**Figure 7:**
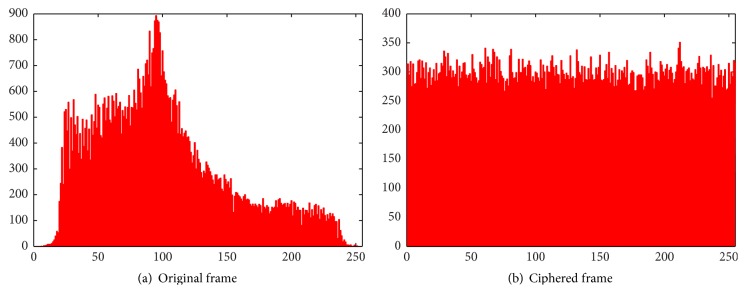
Histograms of red component.

**Figure 8:**
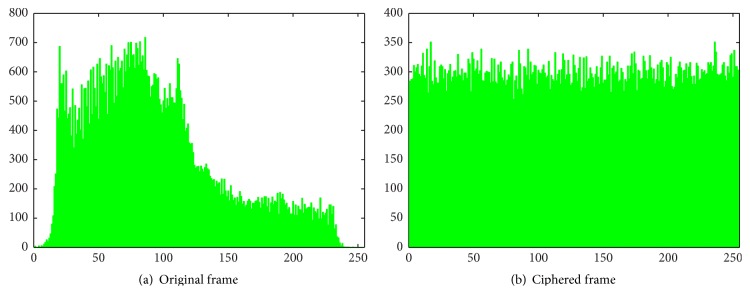
Histograms of green component.

**Figure 9:**
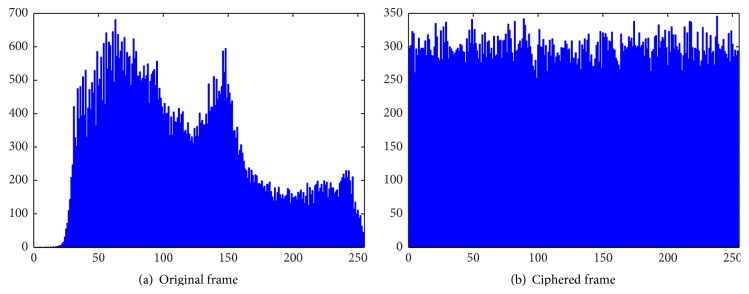
Histograms of blue component.

*(B) Histogram Analysis*. Statistical attacks are made by exploiting the predictable relationships between the plain and cipher frame pairs. Significant changes in the histograms of original and cipher frames reveal the strength of the algorithm against statistical attacks. The histograms of red, green, and blue components of original and ciphered frames are illustrated in Figures 7, 8, and 9, respectively. Since the histograms of the ciphered frame are uniformly distributed and significantly different from the original frames, they do not provide any clue to deploy statistical attacks.

#### 4.2.3. Randomness Test

The National Institute of Standards and Technology (NIST) has recommended certain set of statistical tests for detecting deviations of a binary sequence from randomness [26]. The randomness testing is required in many cryptographic, modelling, and simulation applications.  Table 5 shows the results of the statistical test on the generated binary sequence. The sequence is said to exhibit random behaviour if the value obtained in each of the statistical tests is greater than or equal to 0.01. The result clearly shows that the values obtained for all the tests are greater than 0.01 and hence the proposed design has passed the randomness testing.

## 5. Overall Performance Analysis of Henon Maps in Video Encryption

The performance metrics for video encryption such as visual degradation, trade-off between encryption ratio and speed, compression friendliness, and format compliance are examined in both applications of Henon maps, namely, (i) integration of proposed S-box design with DES and Blowfishand (ii) generation of random keys for simple substitution cipher in counter mode of operation.

### 5.1. Visual Degradation

Visual degradation measures the perceptual distortion of the video data with respect to the plain video. It can be inferred from Figures 5 and 6 that the proposed methodology on integration with symmetric algorithms offers high visual degradation and hence can be used for encrypting sensitive data. As a miniature substitution cipher, the algorithm provides high degradation at a less computational time.

### 5.2. Encryption Ratio versus Speed

Encryption ratio measures the ratio between the size of the encrypted part and the whole data size. In order to increase the speed of the encryption algorithm, encryption ratio has to be minimized. A balance between security and speed is achieved by completely encrypting the entire data and at the same time increasing the speed of encryption through modified design of S-boxes. The proposed design significantly reduces the time taken for encryption/decryption by eliminating the 521 iterations of Blowfish making it suitable for real time processing. In application of the proposed design for generation of random key, the encryption is carried out in counter mode which involves parallel processing of multiple frames for significant reduction in encryption/decryption time. Since every frame is operated independently of the other frame, propagation of bit errors in transmission is completely eliminated and hence the quality of decrypted video is ensured.

### 5.3. Compression Friendliness

The proposed video encryption algorithm is compression friendly as it has no impact on the efficiency of data compression. Also the size of the data is not increased with reference to the original size of the file.

### 5.4. Format Compliance

The syntax of the encrypted video stream of the proposed video encryption algorithm is also compliant with the standardized syntax of the compressed video stream. The encryption does not disturb the standardized syntax structure of the video stream such as the frame header, slice header, and the block header.

## 6. Conclusion and Future Work

This research work deploys the concept of Henon map to create dynamic S-boxes for symmetric key cryptosystems and for generation of random keys for simple substitution ciphers. The randomness, sensitivity to initial conditions, and ergodicity make Henon map suitable for S-box generation and one-time pads. The ease of encoding and transformation technique makes the system suitable for memory limited and low powered devices. The proposed methodology attains a high level of security at a less computational time. Experimental results prove the robustness of the algorithm against statistical attacks, key sensitivity tests, and randomness test and in performance enhancement. Future work may be attempted at deriving mathematical proofs for nonlinearity. The proposed video encryption algorithm may be extended and tested for mobile and cloud environments.

## Figures and Tables

**Table 1:** Sample S-box values for various word lengths.

Word length = 8		Word length = 32	
(*X*_0_ = 0.26, *Y*_0_ = 0.29)	(*X*_0_ = 0.2600001, *Y*_0_ = 0.2900001)	(*X*_0_ = 0.26, *Y*_0_ = 0.29)	(*X*_0_ = 0.2600001, *Y*_0_ = 0.2900001)
187	24	3141672960	411361735
66	132	1089722552	1963237847
32	225	2974290515	3333557337
0	199	745678432	1933694433
64	117	1584790750	450043474
243	4	412521365	2414960418
216	161	1965090619	2698793360
184	215	80970080	25003267
177	198	881337089	1165496663
72	178	1622861053	1773666820

**Table 2:** Analysis of avalanche effect.

Rounds	Output in hexadecimal	
	Input = 0x1234876509874563	Input = 0x1234876509874562
1	27A03F47 A976A765	CD179EFA 243AA986
2	DFCA3C31 6753E7FF	F71F213D 6753E7FE
3	1A8D4140 6E822E62	7407EF0D 4657336E
4	EEE60256 36FF6720	9CEEFEB3 5875C96D
5	8748B200 B093FE88	6B655854 C29B026D
6	ECEAD64C 9FDE2195	2DCBAAAC 73F3CBC1
7	FE41A7F1 99CA3177	A706709E 58EB4D97
8	01A13BCD FA922691	50D35728 A3D5F1FE
9	ACEAD2AC E6F9BACC	702B5C9B B78BD629
10	BEC8D3A8 CC503651	997D1993 1091B866
11	DA02AA08 745FE49C	C7916952 53EA2EA7
12	D49EEFBF 8116B9CB	E5CB473C 9C857A91
13	6EC5BDAD 7832823A	B7E64AD6 49672AB9
14	5BC94D80 291906EA	A17A4A21 F03AF191
15	88E1BB56 F1BF0320	BB7180AF 0B0C0481
16	F15CDE0B5BEADA19	9E85D079 687AE1E0

**Table 3:** Properties of “dfs.avi”.

Attribute	Value
Number of frames	102
Height	240 pixels
Width	320 pixels
Frame rate	15 fps
Bits per pixel	24 bpp
Size of video	1.0839 MB
Video format	RGB24

**Table 4:** Correlation coefficient.

Frame number	Frame number	Correlation coefficient of red component		Correlation coefficient of green component		Correlation coefficient of blue component	
		Before encryption	After encryption	Before encryption	After encryption	Before encryption	After encryption
1	2	0	−0.0065	0	−0.0066	0	−0.0020
2	2	1	1	1	1	1	1
2	3	0.8089	−0.0074	0.7937	−0.0067	0.8211	0.0089
3	4	0.7958	0.00022	0.7744	0.0037	0.7946	0.0087
4	5	0.7799	0.0014	0.7631	0.0036	0.7728	0.00087

**Table 5:** Randomness testing.

NIST statistical tests	Results
Frequency (Monobit) test	0.8597
Runs test	0.7234
Discrete Fourier transform	0.1048
Block frequency	0.8233
Longest runs test	0.0103
Cusum test	0.6547
Serial test	0.7770
Matrix rank test	0.5339
Overlapping template test	0.9060
Linear complexity test	0.9428
Nonoverlapping template test	0.6608
Random excursions variant test	0.6681
Random excursions test	0.6920
